# Ultrasensitization: Switch-Like Regulation of Cellular Signaling by Transcriptional Induction

**DOI:** 10.1371/journal.pcbi.0010054

**Published:** 2005-10-28

**Authors:** Stefan Legewie, Nils Blüthgen, Reinhold Schäfer, Hanspeter Herzel

**Affiliations:** 1 Institute for Theoretical Biology, Humboldt University, Berlin, Germany; 2 Laboratory of Molecular Tumor Pathology, Charité, Berlin, Germany; University of Tokyo, Japan

## Abstract

Cellular signaling networks are subject to transcriptional and proteolytic regulation under both physiological and pathological conditions. For example, the expression of proteins subject to covalent modification by phosphorylation is known to be altered upon cellular differentiation or during carcinogenesis. However, it is unclear how moderate alterations in protein expression can bring about large changes in signal transmission as, for example, observed in the case of haploinsufficiency, where halving the expression of signaling proteins abrogates cellular function. By modeling a fundamental motif of signal transduction, the phosphorylation–dephosphorylation cycle, we show that minor alterations in the concentration of the protein subject to phosphorylation (or the phosphatase) can affect signal transmission in a highly ultrasensitive fashion. This “ultrasensitization” is strongly favored by substrate sequestration on the catalyzing enzymes, and can be observed with experimentally measured enzymatic rate constants. Furthermore, we show that coordinated transcription of multiple proteins (i.e., synexpression) within a protein kinase cascade results in even more pronounced all-or-none behavior with respect to signal transmission. Finally, we demonstrate that ultrasensitization can account for specificity and modularity in the regulation of cellular signal transduction. Ultrasensitization can result in all-or-none cell-fate decisions and in highly specific cellular regulation. Additionally, switch-like phenomena such as ultrasensitization are known to contribute to bistability, oscillations, noise reduction, and cellular heterogeneity.

## Synopsis

Hormones and other external stimuli induce cellular transitions such as cell division or differentiation by regulating gene expression. Hormone-induced cellular transitions are known to occur in a switch-like fashion: while weak background stimuli are rejected, cellular transitions proceed fully as soon as a threshold hormone concentration is exceeded. Earlier studies have described several mechanisms whereby such a switch-like behavior can be realized in intracellular communication via signal transduction networks, which convert hormonal signals into alterations in gene expression.

The authors demonstrate how switch-like behavior can be further enhanced downstream of hormone-induced gene expression. They show that even minor (hormone-induced) alterations in gene expression can dramatically affect the activity of intracellular signal transduction networks, and thereby modify cellular behavior. This phenomenon has been termed “ultrasensitization.”

Ultrasensitization can explain the pronounced dosage sensitivity observed for many disease-associated signal transduction proteins: for example, the mutation of one of two alleles (gene copies), resulting in a 2-fold reduction of gene expression, can already initiate disease progression. Although such sensitivity towards mutations is potentially harmful, the fact that cells nevertheless exhibit ultrasensitization suggests that somehow cells benefit from ultrasensitization. The authors illustrate how ultrasensitization improves the specificity and efficiency of cell-to-cell communication and contributes to cellular memory.

## Introduction

**Figure 1:**
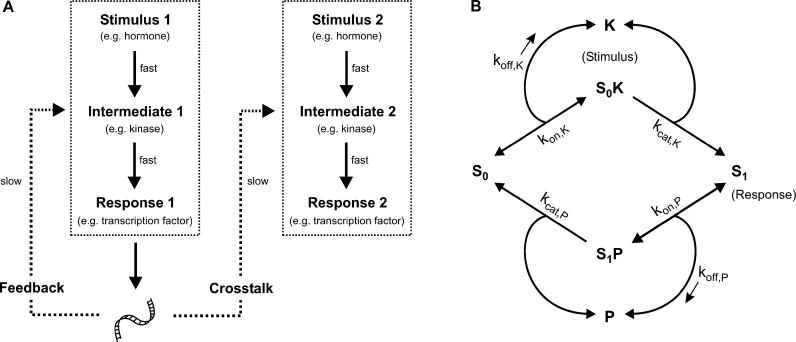
Slow and Fast Regulation of Cellular Signal Transduction (A) Schematic representation of cellular signal transduction. Upstream stimuli (e.g., hormones) result in altered gene expression by eliciting rapid intracellular responses such as transcription factors (fast regulation). The resulting changes in protein expression often in turn affect cellular signal processing of upstream inputs via transcriptional feedback or crosstalk (slow regulation). (B) Schematic representation of a phosphorylation–dephosphorylation cycle, where the kinase K and the phosphatase P catalyze the (de)phosphorylation of the substrate, S. Hormonal stimulation (i.e., fast regulation) was modeled by altering the total kinase concentration, K_tot_ = K + S_0_K, and the steady-state concentration of free phosphorylated substrate, S_1_, was taken as the response. The impact of slow regulation was modeled by varying the concentration of the substrate (S_tot_ = S_0_ + S_0_K + S_1_ + S_1_P) or that of the phosphatase (P_tot_ = P + S_1_P).

Cellular signal transduction exhibits two layers of regulation: upstream stimuli such as extracellular peptide hormones activate intracellular signaling intermediates (e.g., mitogen-activated protein kinases), which in turn induce intracellular responses (e.g., activated transcription factors) as indicated in Figure 1A. This type of regulation, which usually operates on the time scale of minutes, will be termed “fast regulation” in this paper. Fast regulation involves the posttranslational modification of pre-existing protein pools in order to transduce signals to the nucleus. Responses induced by fast regulation (Response 1), such as activated transcription factors, often in turn alter the total abundance of their own activators (Intermediate 1) or that of intermediates in heterologous cascades (Intermediate 2), e.g., owing to induced mRNA/protein synthesis or to degradation (Figure 1A). For example, signal transduction pathways in the immune system alter the concentration of their own constituents by transcriptional positive or negative feedback to bring about sensitization [1] or desensitization [2]. Likewise, cyclic guanosine monophosphate signaling affects the heterologous mitogenic cascades via “transcriptional crosstalk” by inducing the MKP-1 phosphatase and cyclin-dependent kinase inhibitors [3]. Owing to the long half-lives of most mRNAs [4] and proteins [5], this type of regulation operates on the time scale of hours in most (but not all) cases and thus will be referred to as “slow regulation” here.

Figure 1A shows a simplified view of signal transduction: upstream stimuli such as hormones (i.e., fast regulation) induce gene expression (i.e., slow regulation), which in turn alters cellular signal processing. Previous theoretical and experimental studies (e.g., [6,7]) have mainly analyzed how fast regulation influences slow regulation, while downstream effects, i.e., the impact of slow regulation on fast regulation, are less well investigated. However, slow regulation is probably equally important, since the expression of phosphoproteins is altered during a variety of physiological processes such as differentiation [8], development [9], apoptosis [10], long-term potentiation [11], the cell cycle [12], and the circadian rhythm [13]. Furthermore, the deregulated expression of wild-type phosphoproteins has been shown to be correlated with diseases such as diabetes [8] and cancer [14].

At a first glance, one may expect that altered expression of Intermediate 1 (i.e., slow regulation) affects steady-state signal transmission via Intermediate 1 (i.e., fast regulation) in a linear fashion (Figure 1A). However, recent research suggests that strong nonlinearity is observed at least in some cases: a variety of tumor-suppressor genes involved in cellular signal transduction do not follow Knudson's two-hit hypothesis, i.e., they do not require a homozygotic loss of both alleles to support tumor progression. Instead, loss of a single copy, i.e., halving protein expression, is sufficient to abrogate tumor-suppressor function and this phenomenon has been termed haploinsufficiency [15,16]. This suggests that the expression of signaling proteins (i.e., slow regulation) affects signal transmission (i.e., fast regulation) in a highly switch-like fashion. As increased signal transmission elicited by transcriptional induction has been referred to as sensitization [1], such ultrasensitive regulation will be referred to here as “ultrasensitization.”

Available experimental data suggest that ultrasensitization is physiologically advantageous: receptor-tyrosine kinases, which elicit different cellular responses, are known to induce broadly overlapping sets of immediate early genes, although the amplitude of the immediate early gene-induction is receptor-specific [17,18]. Ultrasensitization allows cells to discriminate such minor differences in immediate early gene expression (i.e., in slow regulation), and thus confers specificity to receptor-tyrosine kinase signaling. In addition, even saturating hormone concentrations induce/repress the vast majority of target mRNAs less than 10-fold (e.g., [19]). Thus, the stimulus-response of hormone-induced transcription exhibits significant basal activation, so that mRNA induction (i.e., slow regulation) is relatively insensitive towards extracellular hormone concentrations [20]. In such cases, ultrasensitization can dramatically increase the cellular effects of extracellular hormone administration and may help to establish all-or-none cell-fate decisions. Finally, strong nonlinearities such as ultrasensitization are known to contribute to bistability, oscillations, noise reduction, and cellular heterogeneity (see Discussion).

Phosphorylation is the most common mode of eukaryotic information transfer, and it has been estimated that one third of all cellular proteins are phosphorylated [21]. As several haploinsufficient tumor suppressors encode phosphoproteins (e.g., p53, BRCA1, H2AX, and pRb), or phosphatases (PTEN), we were interested in whether ultrasensitization can occur in a simple phosphorylation–dephosphorylation cycle (Figure 1B). We show here that pronounced ultrasensitization is possible with experimentally measured kinetic constants, particularly in the parameter range where the substrate–enzyme ratio is such that the majority of the substrate subject to (de)phosphorylation is sequestered by the catalyzing enzymes. Furthermore, we show that coordinated transcription of multiple proteins (i.e., synexpression) within a kinase cascade can result in even more pronounced ultrasensitization. Finally, we demonstrate that ultrasensitization can account for specificity and modularity in the regulation of cellular signal transduction.

## Results

### Ultrasensitization in a Phosphorylation–Dephosphorylation Cycle

As outlined in the Introduction, we were interested in the means by which altered protein expression (i.e., slow regulation) affects signal transmission (i.e., fast regulation) by a simple phosphorylation–dephosphorylation cycle depicted in Figure 1B. Protein (de)phosphorylation was modeled using the irreversible Michaelis-Menten mechanism, where each elementary step is described by mass-action kinetics. As indicated in Figure 1B, the free unphosphorylated substrate, S_0_, reversibly associates with the free kinase, K, and the resulting kinase–substrate complex, S_0_K, may irreversibly form the free phosphorylated substrate, S_1_, thereby recycling the free kinase, K. Likewise, the free phosphorylated substrate, S_1_, is subject to dephosphorylation by the phosphatase and this occurs via formation of the substrate–phosphatase complex, S_1_P (see Protocol S1 for the differential equations). Importantly, our modeling approach takes substrate sequestration on the enzyme–substrate complexes, S_0_K and S_1_P, into account, which is in contrast to the well-known Michaelis-Menten approximation for enzyme catalysis, where the substrate concentration is assumed to significantly exceed that of the enzyme. The impact of upstream stimuli such as extracellular hormones (i.e., fast regulation) was modeled by altering the total kinase concentration, K_tot_ = K + S_0_K, and the steady-state concentration of the free phosphorylated substrate, S_1_, was taken as the response of the system. As we assume that the phosphatase competes with downstream effectors for a single docking site in the substrate (e.g., [22]), the substrate–phosphatase complex, S_1_P, does not contribute to the response.

**Figure 2:**
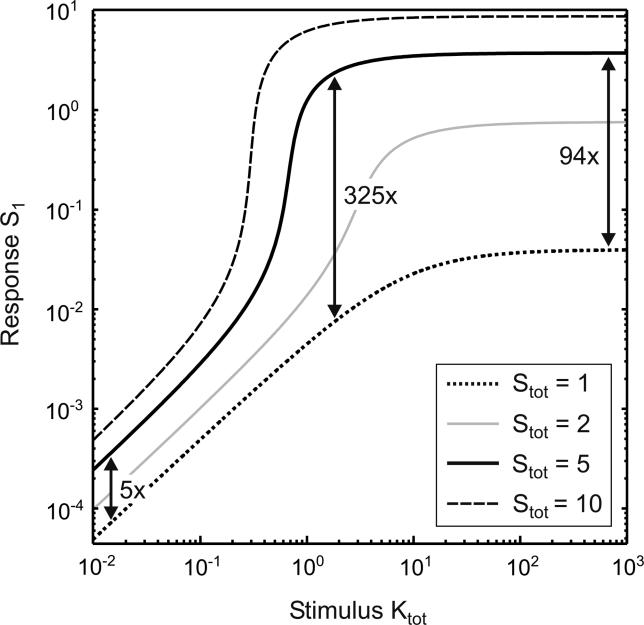
Ultrasensitization in a Phosphorylation–Dephosphorylation Cycle Stimulus-response of the phosphorylation–dephosphorylation cycle depicted in Figure 1B for varying substrate expression levels, S_tot_ = S_0_ + S_0_K + S_1_ + S_1_P, on a double-logarithmic scale. The relative alterations in the response, S_1_, for a given stimulus, K_tot_ = K + S_0_K, elicited by a 5-fold change in substrate expression are indicated next to the vertical arrows. Parameters chosen: k_on,K_ = k_off,P_ = k_cat,P_ = 0.01; k_off,K_ = k_cat,K_ = 1; k_on,P_ = 1.6; P_tot_ = 1.25.

To understand how protein expression (i.e., slow regulation) affects signal transmission (i.e., fast regulation), the steady-state stimulus response of the model depicted in Figure 1B was plotted for varying substrate expression, S_tot_ = S_0_ + S_0_K + S_1_ + S_1_P. Then, the steady-state activation levels (S_1_) for a given stimulus (K_tot_) before and after induction of the substrate (S_tot_) were compared. As indicated by the right arrow in Figure 2, a 5-fold induction of the substrate expression can bring about a 94-fold increase in the steady-state activation level upon strong stimulation (K_tot_ >> P_tot_). Ultrasensitization is even more pronounced upon intermediate stimulation, since then a 5-fold altered substrate expression can bring about a 325-fold increase in the steady-state activation level. By contrast, nonlinearity disappears for very weak stimulation, i.e., for K_tot_ → 0, where the absolute activation levels are negligible (Figure 2).

Thus, we proved that protein expression (i.e., slow regulation) can affect signal transmission (i.e., fast regulation) in a highly nonlinear fashion provided that stimulation is sufficiently strong. In other words, ultrasensitization is possible in a phosphorylation–dephosphorylation cycle, and transfection experiments in living cells support this conclusion [23].

### Ultrasensitization Due to Substrate Sequestration

To gain further insight into ultrasensitization in a phosphorylation–dephosphorylation cycle, we analyzed steady-state signal transmission upon strong stimulation (K_tot_ >> P_tot_), since an analytical expression for the response, S_1_, could be obtained in this limit (Protocol S1):


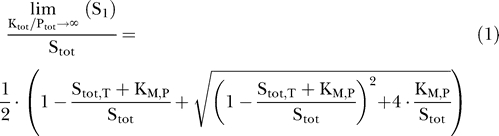


Here, the Michaelis-Menten constant of the phosphatase, K_M,P_, and the threshold substrate concentration, S_tot,T_, where ultrasensitization occurs (see below), are given by:


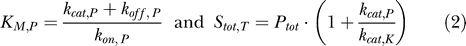


The rate constants k_on_, k_off_, and k_cat_ describe the individual steps of kinase (K) or phosphatase (P) catalysis as indicated in Figure 1B, and P_tot_ = P + S_1_P is the total phosphatase expression level.

**Figure 3:**
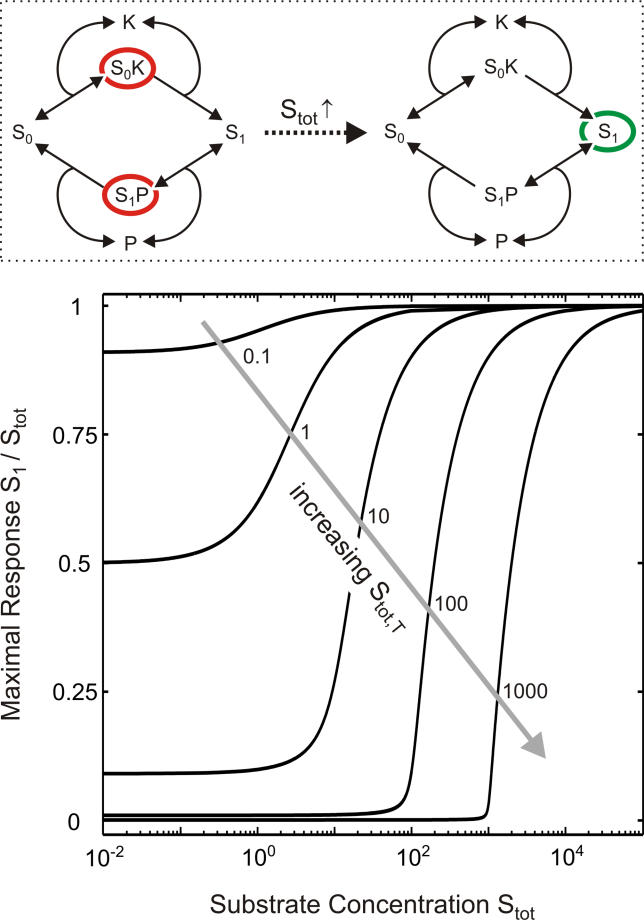
Ultrasensitization Due to Substrate Sequestration The normalized maximal response of the phosphorylation–dephosphorylation cycle depicted in Figure 1B is plotted as a function of substrate expression on a semilogarithmic scale for the limit of strong stimulation (according to Equation 1), where K_tot_ >> P_tot_. The threshold, S_tot,T_, (see Equation 2) was varied as indicated, while the Michaelis-Menten constant of the phosphatase, K_M,P_, was kept constant and assumed to be unity. The scheme on the top indicates the mechanism of ultrasensitization: for weak substrate-expression, most of the substrate is sequestered on the enzyme–substrate complexes, S_0_K and S_1_P, while signal transmission via S_1_ occurs as soon as substrate expression, S_tot_, exceeds the threshold, S_tot,T_.

As shown in Figure 3, a large relative change in signal transmission according to Equation 1 from S_1_ << S_tot_ to S_1_ ≈ S_tot_ can be observed upon minor increases in substrate expression. As this dramatic relative change occurs in addition to the absolute increase in S_tot_, substrate expression, S_tot_ (slow regulation), affects signal transmission via S_1_ (fast regulation) in a highly ultrasensitive fashion (ultrasensitization), particularly if:


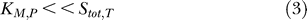


If this condition holds, signal transmission is negligible (i.e., S_1_ << S_tot_) for weak substrate-expression levels, S_tot_ < S_tot,T_ (Figure 3), since then virtually all substrate is sequestered on the enzyme–substrate complexes, S_0_K and/or S_1_P (Protocol S1). By contrast, signal transmission begins to rise in an ultrasensitive fashion (ultrasensitization) as soon as the substrate expression exceeds the threshold, i.e., as soon as S_tot_ > S_tot,T_. This can be explained as follows: the threshold, S_tot,T_, equals the maximal amount of substrate that can be sequestered on the enzyme–substrate complexes, S_0_K and/or S_1_P (Protocol S1), so that substrate sequestration no longer prevents signal transmission if S_tot_ > S_tot,T_. Ultrasensitization finally vanishes for very strong substrate expression (S_tot_ >> S_tot,T_), since then the amount of sequestered substrate is negligible, so that substrate expression affects signal transmission in a linear fashion (i.e., S_1_ ≈ S_tot_).

Thus, we showed that pronounced ultrasensitization due to substrate sequestration on the catalyzing enzymes requires Equation 3 to be fulfilled (Figure 3). As outlined in the Discussion, Equation 3 is in accordance with experimentally measured data, so that ultrasensitization due to sequestration is expected to be observed in vivo. It should be noted that numerical studies revealed that ultrasensitization due to substrate sequestration under the regime of Equation 3 does *not* require very strong stimulation (K_tot_ >> P_tot_), which was assumed to derive analytical expressions, but rather can be observed provided that (Protocol S1):


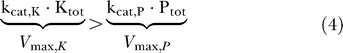


Equation 4 can be considered to be the general requirement for *any* ultrasensitization to occur in a phosphorylation–dephosphorylation cycle, since otherwise kinase activity (i.e., the stimulus) is too weak to elicit significant accumulation of the active species, S_1_. As outlined above, ultrasensitization refers to a large relative increase in signal transmission as the substrate expression level is increased, and thus requires that the majority of substrate is active (i.e., that S_1_ ≈ S_tot_) for sufficiently large S_tot_. For such high substrate expression levels, substrate sequestration on the catalyzing enzymes is negligible, and the overall velocities of phosphorylation (S_0_ → S_1_) and dephosphorlyation (S_1_ → S_0_) can be approximated by V_max,K_ and V_max,P_. According to Goldbeter and Koshland [24], strong signal transmission (i.e., S_1_ ≈ S_tot_) for large S_tot_, and thus ultrasensitization, can be observed only if the kinase velocity exceeds that of the phosphatase, i.e., if Equation 4 holds (Protocol S1). This result confirms our earlier observation that ultrasensitization vanishes upon weak stimulation (see Figure 2).

### Ultrasensitization Due to Activity Switching

Even though ultrasensitization due to substrate sequestration is preserved for intermediate stimulus levels (see above), this does not explain why ultrasensitization can be more pronounced for intermediate stimuli when compared to strong stimulation (Figure 2). For intermediate stimulus levels, an additional mechanism, which is independent of substrate sequestration, can bring about enhanced sensitization. Provided that Equation 4 holds, increasing substrate expression induces an “activity switch” from high overall phosphatase activity (S_1_ → S_0_) to high overall kinase activity (S_0_ → S_1_) if kinase catalysis is significantly less saturated than phosphatase catalysis, i.e., if:


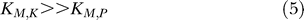


As already mentioned, ultrasensitization refers to a large relative increase in signal transmission as the substrate expression level is increased. While Equation 4 is required for strong signal transmission (S_1_ ≈ S_tot_) for large S_tot_ (see above), Equation 5 ensures that signal transmission vanishes (S_1_ << S_tot_) if S_tot_ is small: for low substrate expression levels, where enzyme saturation is negligible, the overall phosphorylation and dephosphorylation velocities at steady state can be approximated by linear kinetics with the first-order rate constants V_max,K_/K_M,K_ and V_max,P_/K_M,P_ (Protocol S1). If Equations 4 and 5 hold, the phosphatase activity outnumbers that of the kinase (i.e., V_max,P_/K_M,P_ >> V_max,K_/K_M,K_) provided that the stimulus, K_tot_, is not too strong, so that signal transmission does not occur (S_1_ << S_tot_). Hence, a pronounced relative change from weak to strong signal transmission (ultrasensitization) can be observed as the substrate expression level is increased if both Equations 4 and 5 hold. Importantly, this ultrasensitization due to activity switching is independent of substrate sequestration.

**Figure 4:**
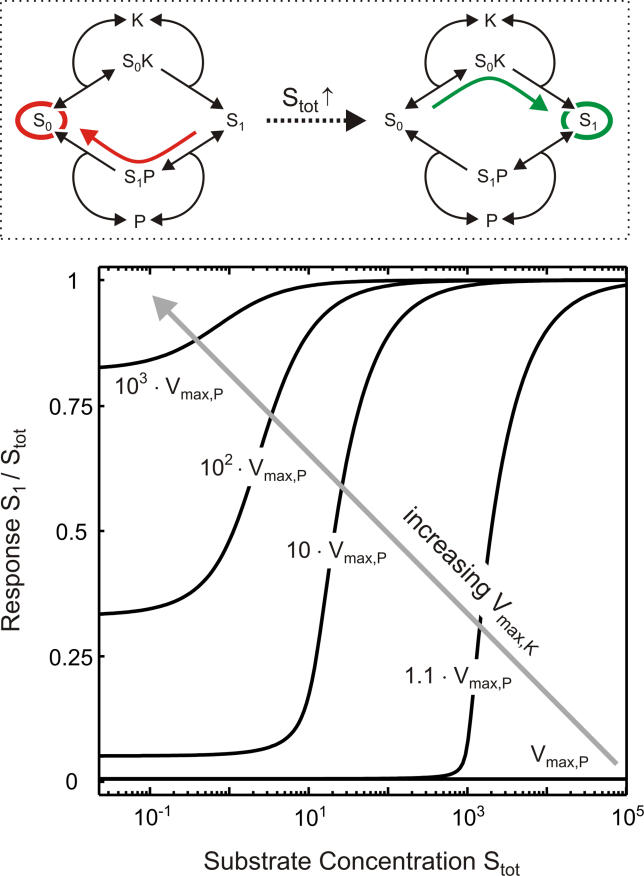
Ultrasensitization Due to Activity Switching The normalized response of the phosphorylation–dephosphorylation cycle depicted in Figure 1B is plotted as a function of substrate expression on a semilogarithmic scale for varying stimulus levels. To relate the plot to analytical results given in the main text (Equation 4), the stimulus, K_tot_, is expressed as V_max,K_ and given in times of V_max,P_. For the parameters chosen (k_on,K_ = 0.02; k_off,K_ = k_cat,K_ = k_off,P_ = 1; k_on,P_ = 2; k_cat,P_ = P_tot_ = 0.1), substrate sequestration is insignificant (i.e., Equation 3 does *not* hold) and the kinase is significantly less saturated than the phosphatase (Equation 5). The scheme on the top indicates the mechanism of ultrasensitization: increasing substrate expression induces a switch from high overall phosphatase activity (S_1_ → S_0_) to high overall kinase activity (S_0_ → S_1_).

Corresponding numerical results are shown in Figure 4 for a phosphorylation–dephosphorylation cycle with weak substrate sequestration, where intermediary stimulus V_max,K_ results in pronounced ultrasensitization due to activity switching. Ultrasensitization disappears for stronger stimulus levels, since then the overall kinase activity outnumbers that of the phosphatase regardless of the substrate expression level (see above). In other words, ultrasensitization due to activity switching is restricted to intermediate stimulus levels, which explains why ultrasensitization in Figure 2 is optimal upon intermediate stimulation.

### Ultrasensitization Due to Synexpression within a Kinase Cascade

**Figure 5:**
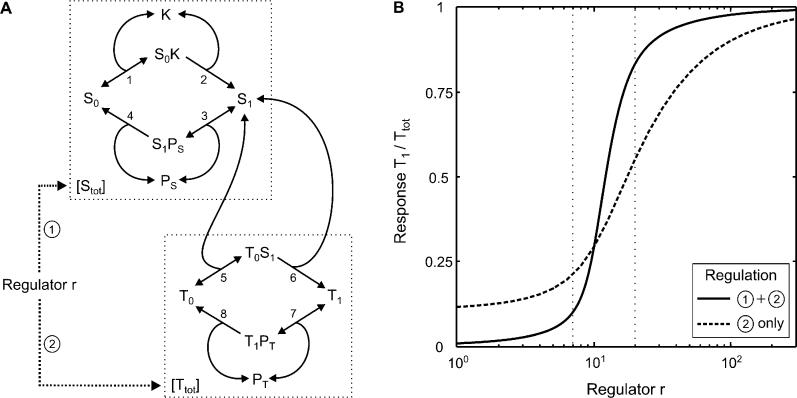
Ultrasensitization Due to Synexpression within a Signaling Cascade (A) Schematic representation of a signaling cascade subject to synexpression. An increase in the regulator, r, was assumed to result in a proportional increase in the expression of intermediates S and T, both of which are subject to covalent modification by (de)phosphorylation. (B) Ultrasensitization due to synexpression within a signaling cascade measured numerically by plotting the normalized response as a function of the regulator concentration, r, where S_tot_ = T_tot_ = r (solid line). To show that synexpression enhances ultrasensitization, the case where the regulator, r, affects transcription of T only is also shown for S_tot_ = 10 (dashed line). Similar results were obtained for other values of S_tot_ or other stimulus strengths (data not shown). Parameters chosen: k_off,1_ = k_off,5_ = k_cat,2_ = k_cat,6_ = k_off,3_ = k_off,7_ = k_cat,8_ = P_S,tot_ = P_T,tot_ = 1; k_on,1_ = 0.02; k_on,5_ = 0.2; k_on,3_ = 2.1; k_on,7_ = 2; k_cat,4_ = 1.1; K_tot_ = 10.

Often multiple intermediates in a signaling cascade of phosphorylation–dephosphorylation cycles (Figure 5A) are transcribed coordinately in a so-called synexpression group. For example, the insulin receptor and its downstream substrate, IRS-1, are both upregulated during adipocyte differentiation [8,25], and this dramatically enhances insulin sensitivity in adipocytes when compared to fibroblastoid precursors. In addition, multiple components of the yeast pheromone-sensing pathway are coordinately induced in a transcriptional positive feedback loop following pheromone stimulation [19,26].

As the impact of such synexpression groups on signal transmission has not yet been investigated, we analyzed a minimal model, where a transcriptional regulator, r, simultaneously induces the expression of the protein S and its downstream substrate, T, both of which are subject to covalent modification by phosphorylation (Figure 5A). For simplicity, we assumed that an increase in the regulator, r, results in a proportional increase in the expression levels of both cascade intermediates, i.e., in S_tot_ and T_tot_. Numerical simulations of the model depicted in Figure 5A demonstrate that synexpression of S and T (solid line in Figure 5B) significantly enhances ultrasensitization when compared to expression of T alone (dashed line in Figure 5B). For example, a 3-fold increase in the regulator, r, (indicated by vertical dotted lines) results in a 24-fold increase in the *non*-normalized response, T_1_, if synexpression is assumed, while only a 9-fold increase is observed in a system devoid of synexpression (Figure 5B). It should be noted that this enhanced sensitization in Figure 5B does *not* result from zero-order ultrasensitivity [24] of the response, T_1_, with respect to intermediate S_1_, but rather is an inherent property of cascades subject to synexpression.

The latter conclusion could be confirmed analytically for a simplified kinase cascade model, where substrate sequestration and enzyme saturation were neglected (Protocol S2). These analytical studies (Protocol S2) revealed that ultrasensitization due to synexpression is observed regardless of the parameters chosen, although the degree of ultrasensitivity is parameter-dependent. As expected, the more cascade stages are coordinately affected by the transcriptional regulator, r, the more pronounced is ultrasensitization due to synexpression (Protocol S2). Because the absolute levels of phosphoproteins within kinase cascades often differ substantially, i.e., S_tot_ ≠ T_tot_ in Figure 5A [27,28], we were interested in how such differential expression affects ultrasensitization due to synexpression. It turned out that ultrasensitization due to synexpression is most pronounced if the absolute concentrations increase along the cascade, i.e., if S_tot_ < T_tot_ in Figure 5A (Protocol S2), as previously reported for the mitogen-activated protein kinase cascade [27,28].

Our modeling studies presented in this section explain why coordinated upregulation of the insulin receptor and its downstream substrate, IRS-1, during adipocyte differentiation results in dramatically enhanced insulin sensitivity (see above). Importantly, ultrasensitization due to synexpression is not restricted to the regulatory mode depicted in Figure 5A, but is also observed if multiple deactivators of a signaling cascade are synexpressed (Protocol S2) as, for example, observed in fibroblast growth-factor signaling pathways [29]. Available experimental evidence supports ultrasensitization due to synexpression, since titration with pervanadate, a general inhibitor of protein tyrosine phosphatases, increases Mek phosphorylation in an ultrasensitive fashion [30], most likely by simultaneously activating multiple tyrosine-phosphorylated proteins including receptors, adaptors, and Mek itself.

### Ultra(de)sensitization Can Bring About Specificity and Modularity

**Figure 6:**
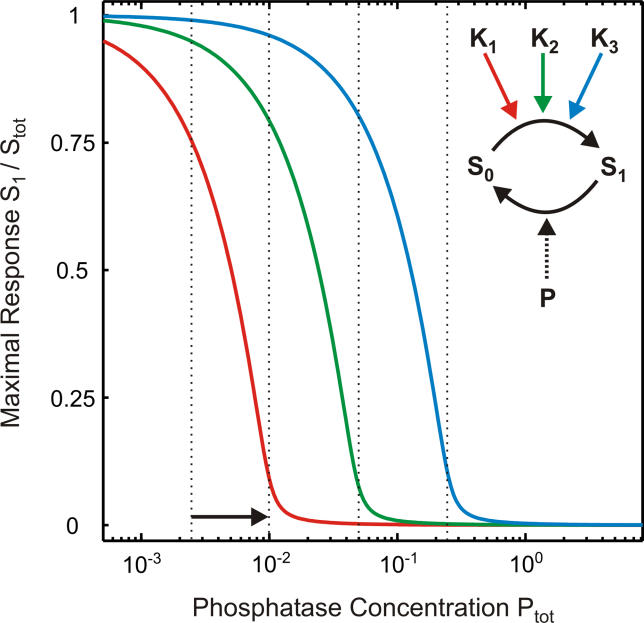
Ultra(de)sensitization Can Bring About Specificity and Modularity The response of a phosphorylation–dephosphorylation cycle (see Figure 1B) upon strong stimulation is shown as a function of the phosphatase expression level (P_tot_ = P + S_1_P) for varying the ratio of the turnover numbers, k_cat,P_/k_cat,K_. Equation 1 was used for plotting, because this expression also applies independently of the relative kinase and phosphatase expression levels provided that K_tot_ >> S_tot_ and K_tot_ >> K_M,K_ (see Protocol S1). The plots shown correspond to the scheme depicted in the upper-right corner, where the free unphosphorylated substrate, S, is phosphorylated by the three kinases, which differ in their turnover numbers, k_cat,K_: K_1_ (red line; k_cat,P_/k_cat,K_ = 100), K_2_ (green line; k_cat,P_/k_cat,K_ = 20), and K_3_ (blue line; k_cat,P_/k_cat,K_ = 3). As indicated by the vertical dotted lines, ultradesensitization may result in four binary regulatory states depending on the phosphatase expression level, particularly if the phosphatase is strongly saturated with its substrate (Equation 6), which is what we assumed here (K_M,P_ = 0.01; S_tot_ = 1).

In the previous sections, we showed that altered substrate expression, S_tot_, can affect signal transmission via phosphorylation–dephosphorylation cycles (see Figure 1B) in a highly ultrasensitive fashion. Likewise, an increase in the phosphatase expression level, P_tot_ = P + S_1_P, can decrease signal transmission upon sufficiently strong stimulation in a switch-like manner (Figure 6) as suggested by transfection studies in living cells [23]. This ultrasensitivity, which will be referred to here as “ultra*de*sensitization,” is particularly pronounced if phosphatase catalysis exhibits strong saturation (see Protocol S1), i.e., if:


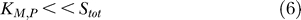


Here, we propose that ultradesensitization due to induced phosphatase expression results in highly specific regulation of cellular signal transmission provided that Equation 6 holds. Consider the scheme depicted in the upper-right corner of Figure 6, where the unphosphorylated substrate, S, is phosphorylated by three kinases, K_1_, K_2_, and K_3_. According to Figure 6, signal transmission via K_1_ (red line) upon strong stimulation is specifically switched off by a relatively minor increase in the phosphatase expression level, P_tot_ = P + S_1_P (indicated by horizontal arrow), while signaling via K_2_ (green line) and K_3_ (blue line) is essentially unaffected. In other words, ultradesensitization due to phosphatase expression specifically switches off individual signaling crosstalk interactions in a binary fashion if Equation 6 holds. The differential sensitivity towards phosphatase expression shown in Figure 6 is due to the fact that catalysis (i.e., k_cat,K_ in Figure 1B) by K_1_ is slower than that by K_2_ and K_3_. Further increasing the phosphatase expression sequentially deactivates signaling via K_2_ and K_3_ as indicated by the vertical dotted lines in Figure 6. Thus, four specific and binary regulatory states can be realized within two orders of magnitude of phosphatase expression, which is the maximal range of transcriptional induction or repression in vivo (e.g., [19]).

Although similar specificity can, in principle, also be achieved by reduced expression of the kinases, K_1_ through K_3_, phosphatase regulation is advantageous for two reasons. First, induced phosphatase expression (as indicated by the vertical arrow in Figure 6) abolishes K_1_-mediated phosphorylation of S, while leaving other actions of K_1_ unaffected, and thereby improves specificity in cellular regulation. Second, transcriptional regulation of phosphatase expression can result in modularization and thereby simplification of cellular regulation: weak phosphatase induction (horizontal arrow in Figure 6) simultaneously downregulates all phosphorylation events that behave like the reaction catalyzed by K_1_ (highly sensitive module), while leaving less sensitive modules (blue and green lines in Figure 6) unaffected.

Importantly, similar conclusions regarding selective and modular regulation of phosphorylation events (shown in Figure 6) also hold if a promiscuous phosphatase dephosphorlyates multiple phosphorylation–dephosphorylation cycles [31–34] with high affinity (Equation 6). In this case, a minor induction in phosphatase expression is predicted to specifically deactivate all highly sensitive phosphorylation–dephosphorylation cycles (red line in Figure 6), while leaving less sensitive cycles essentially unaffected (blue and green lines in Figure 6). Transfection studies support such differential sensitivity of cellular signaling pathways towards phosphatase expression [35,36]. In addition, experimental evidence is consistent with modularization of cellular regulation, since minor alterations in PTB-1B expression were shown to simultaneously affect both cytokine- and insulin-mediated signaling [32].

## Discussion

Previous research on signal transduction has mainly focused on how hormonal stimulation (fast regulation) alters gene expression (slow regulation). In comparison, the impact of gene expression on the processing of hormonal inputs has been less well investigated, although both types of regulation are intimately coupled (see Figure 1A). In the work described here, we used computational methods to show that minor alterations in the expression of signaling proteins can result in large changes in steady-state signal transmission. In other words, we showed that slow regulation of signal transduction can affect fast regulation in a highly nonlinear fashion. Because increased signal transmission elicited by transcriptional induction has been referred to as sensitization [1], we have termed such nonlinearities ultrasensitization.

Experimental data suggest that gene expression is relatively insensitive towards extracellular stimulation (e.g., [19]), so that physiological hormone concentrations will affect cellular protein expression only to a minor extent. Biochemical networks exhibiting ultrasensitization transduce such minor changes into large alterations in cellular function, so that ultrasensitization is expected to enhance the effects of extracellular hormone administration. In addition, subthreshold alterations in protein expression (e.g., S_tot_ < S_tot,T_ in Equation 1) will be neutralized, so that ultrasensitization is predicted to contribute to all-or-none cell-fate decisions.

If the expression of a protein subject to covalent modification by phosphorylation exhibits stochastic variations (reviewed in [37,38]), ultrasensitization can contribute to cellular heterogeneity, which is known to be important in some types of cell differentiation [37]: provided that the mean expression level is in the range of ultrasensitization (i.e., S_tot_ ≈ S_tot,T_ in Equation 1), some cells will be highly sensitive towards extracellular growth factors, whereas others will be essentially insensitive. In a related manner, it has been suggested that increased stochasticity in gene expression contributes to haploinsufficiency of tumor-suppressor genes [39]. In this model, transient reduction of tumor-suppressor function constitutes a window of opportunity for cancer progression. Ultrasensitization might enhance such stochastic effects by transducing relatively minor alterations in gene expression into dramatic changes in tumor-suppressor function. Importantly, ultrasensitization also explains other types of dosage sensitivity, which have been implicated in a variety of diseases. For example, relatively minor overexpression of oncogenic phosphoproteins [14] is predicted to dramatically affect mitogenic signaling, thereby contributing to carcinogenesis. Finally, strong nonlinearities such as ultrasensitization result in bistability or oscillations when combined with positive [40] or negative [41] feedback.

By analyzing steady-state signal transmission for a given stimulus as a function of protein expression, we showed that alterations in substrate expression can bring about ultrasensitization in a phosphorylation–dephosphorylation cycle (see Figure 2) provided that stimulation is sufficiently strong (Equation 4). Importantly, the kinetic scheme depicted in Figure 1B also applies for other types of covalent modification (e.g., acetylation) and for the activation cycles of small G proteins so that ultrasensitization is expected to be a widespread phenomenon in cellular information transfer. Our analytical results demonstrate that ultrasensitization upon strong stimulation requires substrate sequestration on the catalyzing enzymes (Equation 3) to be fulfilled. More specifically, substrate sequestration on the kinase and/or the phosphatase completely prevents signal transmission for weak substrate-expression levels (see Figure 3). As the amount of sequestered substrate cannot exceed the threshold, S_tot,T_ (Equation 2), signal transmission begins to rise in an ultrasensitive fashion (ultrasensitization) as soon as the substrate expression level marginally exceeds the threshold expression level, i.e., if S_tot_ > S_tot,T_ (Figure 3).

Even though transfection experiments suggest that ultrasensitization occurs in vivo [23], we asked whether the predicted requirement for ultrasensitization due to substrate sequestration (Equation 3) is in accordance with available enzyme kinetic data. Unfortunately, Michaelis-Menten constants of phosphatases are often measured using nonphysiological substrates such as *p*-nitrophenyl phosphate (i.e., the Michaelis-Menten constants are likely to be lower in vivo), and only very few quantifications of cellular phosphatase concentrations were performed in particular cell types. Nevertheless, available data reveal some candidate phosphatases such as PP1B (K_M,P_ = 0.04–10 μM; P_tot_ = 0.5 μM) and PP2B (K_M,P_ = 2–20 μM; P_tot_ = 20 μM), whose cellular concentration P_tot_ was reported to exceed the Michaelis-Menten constant K_M,P_ [42]. Likewise, the cellular concentration of PP2A, which has been estimated to be as much as 0.25% of the total cellular protein [43], exceeds the Michaelis-Menten constant K_M,P_ = 10 μM [44].

Even though available data suggest that other phosphatases such as PTP-1B (K_M,P_ = 0.6–8 μM; P_tot_ = 0.02 μM) and PP2C (K_M,P_ = 0.1–0.3 μM; P_tot_ = 0.01 μM) do not fulfill the condition K_M,P_ ≤ P_tot_ [42], it should be kept in mind that subcellular targeting, which has been shown for a variety of phosphatases (e.g., [33,34]), is known to dramatically increase the effective phosphatase concentrations. For example, phosphatases usually exhibit 2- to 10-fold higher concentrations in the nucleus than in the cytoplasm [45]. Likewise, it has been estimated that the effective concentration of signaling proteins is increased by a factor of 1,000 if both the enzyme and the substrate are localized at the membrane [46]. Finally, the threshold S_tot,T_ (i.e., Equation 3) also depends on the ratio of the catalytic rate constants k_cat,P_/k_cat,K_ (see Equation 2), which was experimentally measured for the Erk phosphorylation–dephosphorylation cycle and was shown to be 4.1–23.8 for Erk-dephosphorylation by MKP-3, and even higher (41.3–238.1) for Erk-dephosphorylation by HePTP [47,48]. Thus, experimental data suggest that ultrasensitization due to substrate sequestration occurs in vivo. Accordingly, association of phosphoproteins with catalyzing enzymes was reported to inhibit cellular signal transmission [49–51].

As shown in Figure 2, ultrasensitization can be even more pronounced for intermediate stimulus levels when compared to very strong stimulation. In this case, an additional mechanism, which is independent of substrate sequestration, contributes to pronounced ultrasensitization upon intermediate stimulation (see Figure 4): increased substrate expression induces an ultrasensitive switch from high overall phosphatase activity (S_1_ → S_0_) to high overall kinase activity (S_0_ → S_1_) if Equations 4 and 5 hold true. This ultrasensitization due to activity switching is likely to be physiologically relevant, since it simply requires that kinase catalysis is significantly less saturated than phosphatase catalysis (Equation 5), in addition to sufficiently strong stimulation (Equation 4).

In addition, we proved that coordinated expression of multiple intermediates within a kinase cascade (see Figure 5A) results in ultrasensitization due to synexpression (Figure 5B), which is more pronounced the greater the number of intermediates that are expressed coordinately (see Protocol S2). Available experimental evidence supports ultrasensitization due to synexpression, since titration with pervanadate, a general inhibitor of protein tyrosine phosphatases, increases Mek phosphorylation in an ultrasensitive fashion [30], most likely by simultaneously activating multiple tyrosine-phosphorylated proteins, including receptors, adaptors, and Mek itself. Our modeling studies demonstrate that coordinated expression of multiple cascade proteins, e.g., owing to genomic organization in operons, allows more efficient control over cellular signal transduction, when compared to expression of a single rate-limiting protein. This might be one of the reasons why functionally related proteins are frequently expressed in so-called synexpression groups [52].

Finally, we showed that induced expression of phosphatases also affects cellular information transfer, i.e., fast regulation, via phosphorylation–dephosphorylation cycles (see Figure 1B) in a highly ultrasensitive fashion (ultradesensitization), particularly if phosphatase catalysis is strongly saturated (Equation 6). Importantly, ultradesensitization can account for specificity and modularity in cellular signal transduction: a minor increase in phosphatase expression may coordinately switch off highly sensitive phosphorylation events, which we collectively referred to as the highly sensitive module (red line in Figure 6), while less sensitive modules (green and blue lines in Figure 6) are essentially unaffected. Thus, depending on the phosphatase expression level, cellular signal transduction can exhibit multiple binary (on/off) regulatory states (vertical dotted lines in Figure 6). The modular model of phosphatase action allows highly flexible regulation of cellular signal transduction, since each phosphatase has a characteristic set of substrates, which partially overlaps with that of other phosphatases, so that most phosphoproteins are dephosphorylated by multiple phosphatases [31]. Thus, depending on the phosphatase that is induced transcriptionally, each phosphoprotein might be coregulated with different phosphoproteins; that is, the modular organization differs for each phosphatase.

The paper provides the theoretical framework for more quantitative experimental measurements in order to elucidate the regulation of cellular signal transduction. More specifically, quantitative measurements of how protein expression affects cellular information transfer are highly desirable to understand how haploinsufficiency, specificity, efficiency, bistability, oscillations, noise suppression, and cellular heterogeneity arise. We propose that the predictions made in this paper should be tested experimentally by controlling transcription with recombinant vectors or by the use of transfectants with defined transgene copy numbers. Importantly, it is not sufficient to analyze the phosphorylation status of the protein studied as a measure of signal transmission, since then the potentially inactivating substrate sequestration on the phosphatase (S_1_P-complex in Figure 1B) is not taken into account. Thus, kinase-activity assays like that previously described for Erk activity, which uses Erk-induced MBP-phosphorylation as the readout [53], are needed. We propose to test our predictions experimentally by analyzing the impact of phosphatase expression on Erk-mediated signal transmission for other reasons as well. First, induction of Erk phosphatases owing to transcriptional feedback [29] or crosstalk [3] is known to be physiologically important. Second, the Erk–phosphatase complex (S_1_P in Figure 1B) is known to be catalytically inactive (e.g., [22]), which favors ultrasensitization due to substrate sequestration. Third, ultrasensitization (according to Equation 3) is also likely to be observed owing to the low Michaelis-Menten constant of MKP-3 [48] and to the fact that k_cat,P_ >> k_cat,K_ (see above).

As it might be difficult to achieve coordinated expression of multiple phosphoproteins in transfection experiments, ultrasensitization due to synexpression should be further tested using general inhibitors of kinase or phosphatase action. For example, an experimental investigation on the impact of hypoxia, i.e., oxygen depletion, on cellular survival signaling might be physiologically interesting. Hypoxia results in a dramatic decrease in the cellular adenosine triphosphate concentration from ~1 mM to ~20 μM [54], a value well below the Michaelis-Menten constant for adenosine triphosphate of many kinases, so that the phosphorylation rates of kinases are globally lowered [55,56]. Our analytical results (Protocol S2) predict that survival signaling mediated by protein kinases exhibits a sharp all-or-none response with respect to cellular adenosine triphosphate levels (i.e., the oxygen concentration), so that extracellular survival factors no longer rescue cells from entering into apoptosis as soon as the oxygen concentration falls below a critical level.

Our results also emphasize that the impact of gene expression on hormone-induced signal transduction should be further studied theoretically to gain insight into how other signaling modules respond to altered expression of their constituents. For example, explicit calculations (data not shown) reveal that steady-state signal transmission via proteins subject to multisite phosphorylation is much more sensitive towards induced phosphatase expression when compared to the single-site mechanism assumed in Figure 1B. Such further analyses might be done by using metabolic control analysis (reviewed in [57]), which has been recently applied to understand the impact of gene expression on cellular signaling [58,59]. Signals in living cells are often transient (e.g., owing to receptor downregulation). Although our additional calculations reveal that ultrasensitization is preserved for such transient signals (Protocol S3), further investigations are needed. Interestingly, strong substrate sequestration in a phosphorylation–dephosphorylation cycle (i.e., S_tot_ < S_tot,T_ in Equation 1) does not always completely abolish signal transduction, but results in transient signals owing to adaptation (F. Bruggeman and N. Blüthgen, personal communication), if the rate of kinase catalysis (k_cat,K_) is significantly faster than that for substrate sequestration on the phosphatase (k_on,P_). In this case, ultradesensitization due to altered phosphatase expression mainly affects the signal duration rather than the signal amplitude (data not shown), so that further analysis of ultra(de)sensitization may provide insight into how cells regulate mitogenesis versus differentiation [60].

## Materials and Methods

Numerical simulations were done using the MATLAB computing environment (The Mathworks, Natick, Massachusetts, United States). Analytical results were confirmed using Maple 7 (Waterloo Maple, Waterloo, Ontario, Canada). Computer codes are available from the authors upon request.

## Supporting Information

Protocol S1 Ultrasensitization in a Phosphorylation–Dephosphorylation Cycle—Mathematical Derivations (107 KB PDF)

Protocol S2 Ultrasensitization Due to Synexpression within a Kinase Cascade—Mathematical Derivations (50 KB PDF)

Protocol S3 Ultrasensitization is Preserved upon Transient Stimulation (283 KB PDF)

### Accession Numbers

The Swiss-Prot (http://www.ebi.ac.uk/swissprot) accession numbers for the proteins discussed in this paper are BRCA1 (P38398), Erk (P27361), H2AX (P16104), HePTP (P35236), insulin (P01308), insulin receptor (P06213), IRS-1 (P35568), MBP (P02686), Mek (Q02750), MKP-1 (P28562), MKP-3 (Q16828), p53 (P04637), PP1B (P62140), PP2A (P67775), PP2B (P16298), PP2C (P35813), pRb (P06400), PTB-1B (P18031), and PTEN (P60484).
