# Supplementary material for: Ultrasensitization: Switch-Like Regulation of Cellular Signaling by Transcriptional Induction
Source: PLoS Comput Biol. 2005 Oct 28;1(5):e54. doi: 10.1371/journal.pcbi.0010054 (PMC1274294; doi:10.1371/journal.pcbi.0010054)
Supplement: Protocol S1 — (107 KB PDF) [file pcbi.0010054.sd001.pdf]

S1: Ultrasensitization in a Phosphorylation-Dephosphorylation cycle

S1.1 Ultrasensitization due to substrate sequestration

The differential equations of the phosphorylation-dephosphorylation cycle depicted in Fig. 1B read:

$$\frac{dS_0}{dt} = k_{cat,P} \cdot S_1P - k_{on,K} \cdot S_0 \cdot K + k_{off,K} \cdot S_0K \tag{1.1}$$

$$\frac{dS_0K}{dt} = k_{on,K} \cdot S_0 \cdot K - \left(k_{off,K} + k_{cat,K}\right) \cdot S_0K \tag{1.2}$$

$$\frac{dK}{dt} = \left(k_{off,K} + k_{cat,K}\right) \cdot S_0K - k_{on,K} \cdot S_0 \cdot K \tag{1.3}$$

$$\frac{dS_1}{dt} = k_{cat,K} \cdot S_0K - k_{on,P} \cdot S_1 \cdot P + k_{off,P} \cdot S_1P \tag{1.4}$$

$$\frac{dS_1P}{dt} = k_{on,P} \cdot S_1 \cdot P - \left(k_{off,P} + k_{cat,P}\right) \cdot S_1P \tag{1.5}$$

$$\frac{dP}{dt} = \left(k_{off,P} + k_{cat,P}\right) \cdot S_1P - k_{on,P} \cdot S_1 \cdot P \tag{1.6}$$

Here, k_{on}, k_{off} and k_{cat} are the rate constants for association, dissociation and catalysis. The system exhibits the following mass-conservation relationships for the kinase, the phosphatase and the substrate:

$$K_{tot} = K + S_0K \tag{2.1}$$

$$P_{tot} = P + S_1P \tag{2.2}$$

$$S_{tot} = S_0 + S_0K + S_1 + S_1P \tag{2.3}$$

As indicated in Fig. 1B, the total kinase concentration, K_{tot}, equals the stimulus, while the free phosphorylated substrate, S_1, was assumed to be the response. Even though the differential equation system 1.1 - 1.6 can be reduced from six to three variables by use of the mass-conservation relationships 2.1 - 2.3, no analytical solution could be obtained. Thus, we decided to further simplify the system by assuming strong stimulation (i.e., $K_{tot} >> P_{tot}$). In this case, the free unphosphorylated species, S_0, can be eliminated from the mass-conservation relationship 2.3 as explained in the following.

At steady state, where all derivatives in Eqs. 1.1 - 1.6 equal zero, Eqs. 1.2, 1.5, 2.1 and 2.2 yield:

$$S_0K = K_{tot} \cdot \frac{S_0}{K_{M,K} + S_0} \tag{3.1}$$

$$S_1P = P_{tot} \cdot \frac{S_1}{K_{M,P} + S_1} \tag{3.2}$$

where $K_{M,P} = \frac{k_{cat,P} + k_{off,P}}{k_{on,P}}$ and $K_{M,K} = \frac{k_{cat,K} + k_{off,K}}{k_{on,K}}$. (4)

In addition, Eq. 1.1 can be written as:

$$S_0 = \frac{k_{cat,P} \cdot S_1P + k_{off,K} \cdot S_0K}{k_{on,K} \cdot K}. \tag{5}$$

Substituting this into Eq. 1.2 yields:

$$k_{cat,P} \cdot S_1P = k_{cat,K} \cdot S_0K \tag{6}$$

According to Eqs. 3.1, 3.2 and 6 the free substrate species, S_0 and S_1, are related by:

$$\frac{S_0}{S_1} = \left(\frac{V_{\max,K}}{K_{M,K}} \cdot \frac{K_{M,P}}{V_{\max,P}} + \frac{S_1}{K_{M,K}} \cdot \left(\frac{V_{\max,K}}{V_{\max,P}} - 1 \right) \right)^{-1} \tag{7}$$

where $V_{\max,P} = k_{cat,P} \cdot P_{tot}$ and $V_{\max,K} = k_{cat,K} \cdot K_{tot}$ (8)

It is easy to see from Eq. 7 that the concentration of the free unphosphorylated substrate, S_0, is vanishingly small when compared to that of the free phosphorylated substrate, S_1, if stimulation is sufficiently strong (i.e., if $K_{tot} >> P_{tot}$). Thus, the the mass-conservation relationship of the substrate (Eq. 2.3) simplifies to $S_{tot} \approx S_0K + S_1 + S_1P$. By substituting this into Eq. 6 and by considering Eq. 3.2, one derives the normalized steady state of the free phosphorylated substrate (i.e., the response) upon strong stimulaton:

$$\frac{S_{1,\max}}{S_{tot}} = \frac{\lim_{K_{tot}/P_{tot} \to \infty} (S_1)}{S_{tot}} = \frac{1}{2} \cdot \left(1 - \frac{S_{tot,T} + K_{M,P}}{S_{tot}} + \sqrt{\left(1 - \frac{S_{tot,T} + K_{M,P}}{S_{tot}} \right)^2 + 4 \cdot \frac{K_{M,P}}{S_{tot}}} \right). \tag{9}$$

Here, the threshold, $S_{tot,T}$, where ultrasensitization occurs (see below), is given by:

$$S_{tot,T} = P_{tot} \cdot \left(1 + \frac{k_{cat,P}}{k_{cat,K}} \right) \tag{10}$$

As mentioned in the main text, we were interested whether the substrate expression level, S_{tot}, affects the maximal signal transmission, $S_{1,\max}$ (defined in Eq. 9), upon strong stimulation in an ultrasensitive fashion. Fig. 3 in the main text suggests that signal transmission increases with increasing substrate expression in a linear fashion (i.e., $S_{1,\max} / S_{tot} \approx 1$) as long as $K_{M,P} >> S_{tot,T}$, whereas strong ultrasensitivity is observed if

$$K_{M,P} << S_{tot,T}. \tag{11}$$

To confirm that switch-like behaviour of signal transmission with respect to substrate expression ('ultrasensitization') primarily depends on the ratio of the Michaelis constant of the phosphatase, $K_{M,P}$, and the threshold, $S_{tot,T}$, we calculate the response coefficient (i.e., the gain)

$$R_{S_{tot}}^{S_{1,\max}} = \frac{S_{tot}}{S_{1,\max}} \cdot \frac{dS_{1,\max}}{dS_{tot}} \tag{12}$$

by using Eq. 9. This yields:

$$\mathrm{R}_{\mathrm{S_{tot}}}^{\mathrm{S_{1,max}}} = \frac{\mathrm{f} + \mathrm{K_{M,P}} / \mathrm{S_{tot,T}}}{\mathrm{f}^2 + \mathrm{K_{M,P}} / \mathrm{S_{tot,T}}} \tag{13}$$

Here, the activated fraction, $\mathrm{f} \in [0,1]$, is given by:

$$\mathrm{f} = \frac{\mathrm{S_{1,max}} / \mathrm{S_{tot}} - \lim\limits_{\mathrm{X_{tot}} \to 0} \left(\mathrm{S_{1,max}} / \mathrm{S_{tot}} \right)}{\lim\limits_{\mathrm{X_{tot}} \to \infty} \left(\mathrm{S_{1,max}} / \mathrm{S_{tot}} \right) - \lim\limits_{\mathrm{X_{tot}} \to 0} \left(\mathrm{S_{1,max}} / \mathrm{S_{tot}} \right)} \tag{14}$$

Equation 13 confirms that Eq. 11 is a prerequisite for pronounced nonlinearity (i.e., for $R_{S_{tot}}^{S_{1,\max}} >> 1$). Before analyzing this nonlinearity in more detail, we shall discuss the biological meaning of Eq. 11 and that of the threshold, $S_{tot,T}$ (see Eq. 10).

Since low $K_{M,P}$-values refer to high affinity between substrate and phosphatase, we hypothesized that weak signal transmission ($S_{1,max}$ / S_{tot} < 1) under the regime of Eq. 11 results from strong substrate sequestration on one or both the catalyzing enzymes. The amount of substrate sequestered on both enzymes upon strong stimulation (according to Eqs. 3.2 and 6) is given by:

$$S_0K + S_1P = P_{tot} \cdot \left(1 + \frac{k_{cat,P}}{k_{cat,K}} \right) \cdot \frac{S_1}{K_{M,P} + S_1} \overset{S_{tot} \to 0}{\approx} \frac{P_{tot}}{K_{M,P}} \cdot \left(1 + \frac{k_{cat,P}}{k_{cat,K}} \right) \cdot S_1 \tag{15}$$

Thus, Eq. 11 implies that the concentration of the output species, S_1, is vanishingly low when compared to the enzyme-substrate complexes, S_0K and S_1P, at least for weak substrate expression levels, i.e., for $S_{tot} \to 0$ (see Eq. 15). In other words, most of the substrate is sequestered on the kinase and/or the phosphatase. As obvious from Eq. 6, it is the ratio of the catalytic rate constants, $k_{cat,P}$ and $k_{cat,K}$, which determines the relative contribution of kinase and phosphatase to substrate sequestration.

As shown in Fig. 3 substrate sequestration on the catalyzing enzymes disappears for sufficiently strong substrate expression (i.e., $S_{tot} >> S_{tot,T}$) even if Eq. 11 holds, since then $S_{1,max}$ / $S_{tot} \approx 1$. Under the regime of Eq. 11 we can approximate Eq. 9 by

$$\frac{S_{1,\max}}{S_{tot}} \approx \frac{1}{2} \cdot \left(1 - \frac{S_{tot,T}}{S_{tot}} + \left| 1 - \frac{S_{tot,T}}{S_{tot}} \right| \right). \tag{16}$$

Here, no signal transmission (i.e., strong substrate sequestration) is observed if $S_{tot} < S_{tot,T}$, while $S_{1,max}$ / $S_{tot} > 0$ as soon as the substrate expression, S_{tot}, exceeds the threshold. Biologically, $S_{tot,T}$ equals the maximal amount of substrate, which can be sequestered on the substrate-phosphatase ($S_1P \leq P_{tot}$) and the substrate-kinase ($S_0K \leq P_{tot} \cdot k_{cat,P} / k_{cat,K}$; see Eq. 6) complexes. In other words, substrate sequestration exhibits saturation with respect to substrate expression and is insignificant for $S_{tot} >> S_{tot,T}$. As shown in Fig. 3 in the main text, $S_{tot,T}$ gives a good estimate for the threshold, where ultrasensitization is observed, even if Eq. 11 does not hold.

To estimate the degree of ultrasensitization and to get insight for which substrate expression levels ultrasensitization is especially pronounced, we calculate the response coefficient of Eq. 16, which yields:

$$R_{S_{tot}}^{S_{1,max}} \approx \frac{1}{f} \qquad (17)$$

Here, the activated fraction, f, is given by Eq. 16. This result demonstrates that under the regime of Eq. 11 small relative changes in the substrate expression level, S_{tot}, can result in very large relative changes in signal transmission, $S_{1,max}$, since $R_{S_{tot}}^{S_{1,max}}$ may be much greater than unity (see Kholodenko et al., 1997). Especially strong ultrasensitization ($R_{S_{tot}}^{S_{1,max}} >> 1$) is observed near the threshold expression level (i.e., $S_{tot} \approx S_{tot,T}$), where most of the substrate is sequestered on the enzyme-substrate complexes (i.e., $f \approx 0$). By contrast, ultrasensitization disappears if $S_{tot} >> S_{tot,T}$ (i.e., $f \approx 1$), that is, $S_{1,max}$ increases with increasing S_{tot} in a linear fashion ($R_{S_{tot}}^{S_{1,max}} \approx 1$). Similar conclusions also hold if Eq. 13 is analyzed directly.

S1.2 Ultrasensitization requires sufficiently strong stimulation

Numerical analyses (not shown) revealed that ultrasensitization due to sequestration does not require strong stimulation ($K_{tot} >> P_{tot}$), but rather can be observed as long as Eq. 4 in the main text holds. Likewise, ultrasensitization due to activity switching requires Eq. 4 in the main text to be fulfilled. In the following we give an intuitive explanation why Eq. 4 in the main text represents a general requirement for ultrasensitization in a phosphorylation-dephosphorylation cycle.

Ultrasensitization refers to a large relative change from weak responses ($S_1 << S_{tot}$) to strong responses ($S_1 \approx S_{tot}$) in addition to the absoulte increase in the substrate expression level, S_{tot} (see main text). Hence, S_1 must be large (i.e., $S_1 \approx S_{tot}$) for sufficiently large S_{tot}. In the limit $S_{tot} \to \infty$ the concentration of the substrate (S_{tot}) outnumbers those of the catalyzing enzymes (K_{tot} and P_{tot}). In other words, substrate sequestration on the catalyzing enzymes is vanishingly low ($S_0K \approx K_{tot} << S_{tot}$ and $S_1P \approx P_{tot} << S_{tot}$), so that the corresponding complexes (S_0K and S_1P) can be neglected in the mass conservation relationship 2.3. Then, the differential equation system 1.1 - 1.6 reduces to:

$$\frac{dS_1}{dt} = k_{cat,K} \cdot K_{tot} \cdot \frac{S_{tot} - S_1}{K_{M,K} + S_{tot} - S_1} - k_{cat,P} \cdot P_{tot} \cdot \frac{S_1}{K_{M,P} + S_1} \qquad (18)$$

The steady state solution of this expression was explicitely given by Goldbeter and Koshland (1981). Here, we restrict the analysis to the limit $S_{tot} \to \infty$, which yields:

$$\frac{\lim_{X_{tot} \to \infty} (S_1)}{S_{tot}} = \frac{1}{2} \cdot \left(1 + \frac{\left| \left(k_{cat,K} \cdot K_{tot}\right) / \left(k_{cat,P} \cdot P_{tot}\right) - 1 \right|}{\left(k_{cat,K} \cdot K_{tot}\right) / \left(k_{cat,P} \cdot P_{tot}\right) - 1} \right) \qquad (19)$$

Thus, strong signal transmission (i.e., $S_1 = S_{tot}$) in the limit $S_{tot} \to \infty$ can only be observed if

$$\underbrace{k_{cat,K} \cdot K_{tot}}_{V_{max,K}} > \underbrace{k_{cat,P} \cdot P_{tot}}_{V_{max,P}} \qquad (20)$$

while the response vanishes (i.e., $S_1 << S_{tot}$) otherwise. Hence, ultrasensitization necessarily requires Eq. 20 to be fulfilled, i.e., sufficiently strong stimulation, K_{tot}.

S1.3 Ultrasensitization due to activity switching

In this Section we give an intuitive explanation for ultrasensitization due to activity switching and subsequently analyze the observed ultrasensitivity quantitatively for the special case, where both catalyzing enzymes are expressed at vanishingly low levels.

As outlined in Section 1.2, strong signal transmission ($S_1 \approx S_{tot}$) is observed for high substrate expression levels ($S_{tot} \rightarrow \infty$) as long as Eq. 20 holds. Simultaneously, signal transmission can be weak ($S_1 << S_{tot}$) for low substrate expression levels ($S_{tot} << K_{M,K}$) independently of substrate sequestration as long as the stimulus is not too strong ($V_{max,K} \approx V_{max,P}$), since then Eq. 7 simplifies to:

$$\frac{S_0}{S_1} \approx \frac{V_{\max,P}}{K_{M,P}} \cdot \frac{K_{M,K}}{V_{\max,K}} \tag{21}$$

This expression implies weak signal transmission (i.e., $S_1 << S_{tot}$) for $K_{M,K} >> K_{M,P}$ even if Eq. 20 holds, since $S_1 << S_0$. Thus, a switch from weak to strong signal transmission, i.e., from high phosphatase overall velocity to high kinase overall velocity, is observed as the substrate expression level is increased ('ultrasensitization due to activity switching'). It is immediately obvious that this switch is ultrasensitive, since a *relative* increase in signal transmission from $S_1 << S_{tot}$ to $S_1 \approx S_{tot}$ is observed in addition to the obvious absolute increase in S_{tot}. It should be noted that the condition $K_{M,K} >> K_{M,P}$ (together with Eq. 20) *always* results ultrasensitization, but it depends on the parameters chosen which mechanisms participate: If Eq. 11 holds, substrate sequestration is significant ($S_1 << S_0 << S_0K + S_1P$) for weak substrate expression levels, so that both ultrasensitization due to substrate sequestration and ultrasensitization due to activity switching contribute. Otherwise, ultrasensitization due to activity switching ensures that sensitization occurs in a highly switch-like fashion even in the absence of substrate sequestration (see below).

To confirm that ultrasensitization due to activity switching can indeed result in a highly ultrasensitive increase in signal transmission, we analyze a special case, where substrate sequestration is negligible: If one assumes that both catalyzing enzymes are expressed at vanishingly low levels (i.e., $K_{tot} \rightarrow 0$ and $P_{tot} \rightarrow 0$), the systems' dynamics can be approximated by Eq. 18 (see above) even if the substrate expression level is varied. In the case, where Eq. 20 holds, the steady state solution of Eq. 18, which was explicitely given by Goldbeter and Koshland (1981), can be written as:

$$\frac{S_1}{S_{tot}} = \frac{1}{2} \cdot \left(1 - \frac{w \cdot K_{M,P} + K_{M,P}}{(1-w) \cdot S_{tot}} + \sqrt{\left(1 - \frac{w \cdot K_{M,P} + K_{M,P}}{(1-w) \cdot S_{tot}} \right)^2 + 4 \cdot \frac{K_{M,P}}{(1-w) \cdot S_{tot}}} \right) \tag{22}$$

with: $$w = \frac{k_{cat,P} \cdot P_{tot}}{k_{cat,K} \cdot K_{tot}} \tag{23}$$

Note that Eq. 22 is similar to Eq. 9, which demonstrates that ultrasensitization is possible even if substrate sequestration is negligible. To get further insight into the mechanism of ultrasensitization, we calculate the response coefficient (see Eq. 12), which yields:

$$R_{X_{tot}}^{X_1} = \frac{f + K_{M,P} / (w \cdot K_{M,K})}{f^2 + K_{M,P} / (w \cdot K_{M,K})} \tag{24}$$

Here, the activated fraction is given by:

$$f = \frac{S_1/S_{tot} - \lim_{X_{tot}\to 0} (S_1/S_{tot})}{\lim_{X_{tot}\to\infty} (S_1/S_{tot}) - \lim_{X_{tot}\to 0} (S_1/S_{tot})} \tag{25}$$

Since f ≤ 1, strong ultrasensitization, i.e., $R_{S_{tot}}^{S_1} >> 1$, requires that (see Eqs. 20 and 23):

$$\frac{V_{max,P}}{K_{M,P}} >> \frac{V_{max,K}}{K_{M,K}} \tag{26}$$

Together with the necessary condition for ultrasensitization (Eq. 20) this implies:

$$K_{M,K} >> K_{M,P} \tag{27}$$

As expected from the intuitive derivation given above, ultrasensitization requires that the kinase is significantly less saturated than the phosphatase. In accordance with the assumptions to derive Eq. 21, ultrasensitization due to activity switching is restricted to intermediate stimulus level, since Eq. 26 does not hold for very strong stimuli, K_{tot}.

S1.4 Ultradesensitization due to induced phosphatase expression

As mentioned in the main text, increased phosphatase expression can bring about ultradesensitization in a phosphorylation-dephosphorylation cycle (see Fig. 6). Even though the assumption $K_{tot} >> P_{tot}$, which we made to derive Eq. 9, does not necessarily hold if phosphatase expression is altered, Eq. 9 can still be used to analyze ultradesensitization in response to increased phosphatase expression as explained in the following: For Eq. 9 to apply, the free unphosphorylated substrate, S_0, must be negligible small, so that it can be eliminated from the mass-conservation relationship 2.3 (see above). According to Eq. 3.1 the steady state concentrations of S_0 and S_0K are related by:

$$\frac{S_0}{S_0 K} = \frac{K_{M,K} + S_0}{K_{tot}} \tag{28}$$

This demonstrates that S_0 can be eliminated from the mass-conservation relationship 2.3 (i.e., that $S_0K >> S_0$) if $K_{tot} >> K_{M,K}$ and $K_{tot} >> S_{tot}$, i.e., if stimulation is sufficiently strong.

To quantify how minor changes in phosphatase expression affect signal transmission, we calculate the response coefficient of $S_{1,max}$ with respect to P_{tot} (like in Eq. 12) by using Eq. 9. This yields:

$$R_{P_{tot}}^{S_{1,\max}} = -\frac{(1-f)\cdot\left(\frac{K_{M,P}}{S_{tot}}+f\right)}{\frac{K_{M,P}}{S_{tot}}+f^2}. \quad (29)$$

Here, the activated fraction, f, is given by:

$$f = \frac{S_{1,\max}/S_{tot} - \lim\limits_{P_{tot}\to\infty}\left(S_{1,\max}/S_{tot}\right)}{\lim\limits_{P_{tot}\to 0}\left(S_{1,\max}/S_{tot}\right) - \lim\limits_{P_{tot}\to\infty}\left(S_{1,\max}/S_{tot}\right)} = \frac{S_{1,\max}/S_{tot}}{\lim\limits_{P_{tot}\to 0}\left(S_{1,\max}/S_{tot}\right)} \quad (30)$$

As expected, the response coefficient is always negative, since $S_{1,max}$ decreases with increasing phosphatase expression. Obviously, strong ultradesensitization, i.e., $R_{P_{tot}}^{S_{1,max}} << -1$, is observed if the phosphatase is strongly saturated with its substrate, i.e., if $S_{tot} >> K_{M,P}$.

References

Goldbeter A, Koshland DE Jr. (1981) An amplified sensitivity arising from covalent modification in biological systems. Proc Natl Acad Sci U S A 78: 6840-6844.

Kholodenko BN, Hoek JB, Westerhoff HV, Brown GC (1997) Quantification of of information transfer via cellular signal transduction pathways. FEBS Lett 414: 430-434
