# Supplementary material for: Ultrasensitization: Switch-Like Regulation of Cellular Signaling by Transcriptional Induction
Source: PLoS Comput Biol. 2005 Oct 28;1(5):e54. doi: 10.1371/journal.pcbi.0010054 (PMC1274294; doi:10.1371/journal.pcbi.0010054)
Supplement: Protocol S2 — (50 KB PDF) [file pcbi.0010054.sd002.pdf]

S2: Ultrasensitization due to synexpression within a Protein kinase cascade

As mentioned in the main text, we were interested how a global regulator, r (see Fig. 5A), which simultaneously affects the concentrations of multiple signalling intermediates, alters signal transmission through a protein kinase cascade. In order to obtain analytical results, we analyzed a protein kinase cascade, where all phosphorylation and dephosphorylation reactions proceed with linear kinetics. The differential equation describing the phosphorylated intermediate at the i-th cascade stage reads:

$$\frac{dx_i}{dt} = k_{K,i} \cdot x_{i-1} \cdot X_{tot,i-1} \cdot (1 - x_i) - k_{P,i} \cdot x_i \tag{1}$$

Here, $k_{K,i}$, $k_{P,i}$ and $X_{tot,i}$ are the kinase rate constant, the phosphatase rate constant and the total intermediate expression at the i-th cascade stage. The concentration of each phosphorylated intermediate X_i is given in normalized form, i.e.,

$$x_i = \frac{X_i}{X_{tot,i}} \tag{2}$$

As shown by Heinrich et al. (2002), the steady state of any phosphorylated intermediate can be written in Michaelis-Menten form:

$$x_i = \lim_{S \to \infty} (x_i) \cdot \frac{\psi}{\psi + K_{M,i}} = x_{\max,i} \cdot \frac{\psi}{\psi + K_{M,i}} \tag{3}$$

Here, $\psi = X_0$ is the stimulus, which mediates the phosphorylation of the first intermediate, X_1 (see Eq. 1). In the following we will derive analytical expressions for $x_{max,i}$ and $K_{M,i}$ in order to analyze signal transmission for varying levels of the regulator, r. The steady state solution of Eq. 1 reads:

$$x_i = \frac{x_{i-1}}{x_{i-1} + L_i} \text{ with } L_i = \frac{k_{P,i}}{k_{K,i} \cdot X_{tot,i-1}} \tag{4}$$

In the limit of infinitely strong stimulation ($\psi \to \infty$), Eq. 4 can be rewritten as:

$$x_{\max,i} = \frac{x_{\max,i-1}}{x_{\max,i-1} + L_i} \text{ (with } x_{\max,1} = 1\text{)} \tag{5}$$

Using this expression one obtains:

$$x_{\max,i} = \frac{\lim_{S \to \infty}(X_i)}{X_{tot,i}} = \left(1 + \sum_{j=1}^{i-1}\left(\prod_{k=1}^{j} L_{i-k+1}\right)\right)^{-1} \tag{6}$$

By using Eq. 6 and the relationship

$$K_{M,i} = K_{M,i-1} \cdot \frac{L_i}{L_i + x_{\max,i-1}} \text{ (with } L_1 = \frac{k_{P,1}}{k_{K,1}}\text{),} \tag{7}$$

one derives for the half-maximal stimulus level

$$K_{M,i} = x_{\max,i} \cdot \prod_{j=1}^{i} L_j \ . \tag{8}$$

To analyze how the regulator, r, affects signal transmission, we shall assume that the regulator r leads to a proportional increase in the expression of all substrates, i.e., all $X_{tot,i}$. Thus, the local sensitivities, L_i (see Eq. 4), modify to:

$$L_i = \frac{k_{P,i}}{k_{K,i} \cdot X_{tot,i-1}} = \frac{k_{P,i}}{k_{K,i} \cdot K_i \cdot r} = \frac{l_i}{r} \ \text{(with } L_1 = \frac{k_{P,1}}{k_{K,1}}\text{)} \tag{9}$$

Here, K_i equals the first-oder rate constant for protein synthesis divided by that for protein degradation. Plotting the maximal activation level, $x_{max,i}$, and the half-maximal stimulus, $K_{M,i}$, (Eqs. 6 and 8) as a function of the regulator, r, reveals that both respond in an ultrasensitive fashion (not shown). In other words, simultaneous expression of multiple cascade intermediates results in ultrasensitization of signal transduction. To gain further insight into this 'ultrasensitization due to synexpression', we restrict the following analysis to weak ($\psi \rightarrow 0$) and strong ($\psi \rightarrow \infty$) stimulation.

Weak stimulation: Using Eqs. 6 and 8 signal transmission (Eq. 3) upon weak stimulation (i.e., $\psi << K_{M,i}$) can be approximated by:

$$x_i \overset{\psi << K_{M,i}}{\approx} \frac{x_{\max,i}}{K_{M,i}} \cdot \psi = \frac{r^{i-1}}{L_1 \cdot \prod_{j=2}^{i} (l_j)} \cdot \psi \tag{10}$$

It should be noted that signal transmission via X_i is given in normalized form (see Eq. 2), so that the impact of the regulator, r, on the expression of the cascade stage under consideration (i.e., $X_{tot,i}$) cancels out. Thus, Eq. 40 gives an estimate how signal transmission upon weak stimulation ($\psi \rightarrow 0$) is regulated by, r, in addition to the obvious linear increase. Obviously, signal transmission upon weak stimulation is *always* subject to ultrasensitization as soon as two or more cascade stages are synexpressed, since any change in the regulator, r, results in an r^{i-1}-fold alteration in signal transmission in addition to the obvious linear increase.

Strong stimulation: By definition, signal transmission upon strong stimulation is determined by the maximal activation level, $X_{max,i}$ (Eq. 6). For simplicity, we shall restrict the following analysis to the limiting cases $L_{i-1} << L_i$ and $L_{i-1} >> L_i$, which are analogous to negative and positive cooperativity in protein association. If one assumes that all rate constants within the cascade are equal, i.e., $k_{P,i} = k_P$ and $k_{K,i} = k_K$, the limiting cases mean that the expression levels, $X_{tot,i}$, strongly decrease ($L_{i-1} << L_i$) or increase ($L_{i-1} >> L_i$) along the cascade. In the limiting cases Eq. 6 (with i > 1) can be approximated by (see Eq. 9):

$$x_{\max,i} \overset{[L_{i-1} << L_i]}{\approx} \frac{r}{r + l_i} \ \text{and} \ x_{\max,i} \overset{[L_{i-1} >> L_i]}{\approx} \frac{r^{i-1}}{r^{i-1} + \prod_{k=1}^{i-1} l_{i-k+1}} \tag{11}$$

Again, both expressions are given in normalized form to measure nonlinearity (see above). Since the approximation on the right ($L_{i-1} >> L_i$; 'positive cooperativity') has the form of the Hill equation, the regulator, r, alters signal transmission in a highly switch-like fashion, especially if many cascade stages are subject to synexpression (i.e., if the exponent i-1 is

large). By contrast, weaker ultrasensitization is observed in the limit $L_{i-1} << L_i$ ('negative cooperativity'), since the corresponding approximation (see Eq. 11) has the form of the Michaelis-Menten equation. In addition, the degree of nonlinearity then no longer depends on the number of cascade stages simultaneously altered by the regulator, r.

Conclusions: Comparison of Eqs. 11 and 10 reveals that ultrasensitization upon weak stimulation is either equal to or stronger than that observed upon strong stimulation. Further analysis of Eq. 3 reveals that the degree of ultrasensitivity upon intermediate stimulation lies between that observed for the limits of weak and strong stimulation (not shown).

Hence we can conclude that synexpression *always* results in ultrasensitization (Eqs. 10 and 11). In addition, ultrasensitization usually increases the more cascade stages are coordinately affected by the regulator, r. If one assumes that all rate constants within the cascade are equal, i.e., $k_{P,i} = k_P$ and $k_{K,i} = k_K$, ultrasensitization is strongly favoured if the absolute concentrations increase along the cascade, i.e., if $X_{tot,i} >> X_{tot,i-1}$. Finally, it is worth noting that similar conclusions regarding ultra(de)sensitization also hold if multiple phosphatases/deactivators are synexpressed or if multiple kinase rate constants are simultaneously altered (see Eq. 9). Thus, ATP depletion due to hypoxia is predicted to switch off cellular signalling pathways in an all-or-none fashion as soon as the ATP level falls below a critical threshold (see also main text).

References

Heinrich R, Neel BG, Rapoport TA (2002) Mathematical models of protein kinase signal transduction. Mol Cell 9: 957-70.
