# Supplementary material for: Ultrasensitization: Switch-Like Regulation of Cellular Signaling by Transcriptional Induction
Source: PLoS Comput Biol. 2005 Oct 28;1(5):e54. doi: 10.1371/journal.pcbi.0010054 (PMC1274294; doi:10.1371/journal.pcbi.0010054)
Supplement: Protocol S3 — (283 KB PDF) [file pcbi.0010054.sd003.pdf]

S3: Ultrasensitization is Preserved for Transient Stimuli

In the following we show that ultrasensitization is preserved (albeit weakened) upon transient stimulation (e.g. due to receptor downregulation) as long as the stimulus duration is sufficiently long to elicit any signal transmission.

To model transient stimulation, we shall assume that the phosphorylating kinase, K, is a receptor, which is subject to deactivating internalization. Since receptor internalization is usually slow when compared to receptor-ligand association and receptor (de)phosphorylation, the time courses of receptors can be approximated by a decaying exponential (Heinrich et al., 2002). In the models, which were analyzed in the paper, this was implemented by assuming that the free kinase is removed with a first-order rate constant k_{int} (see Fig. S1A and S1B). Hence, we assumed that the substrate, S, and factors which mediate receptor internalization (or deactivating phosphorylation) bind competitively to the kinase.

Unless otherwise mentioned, ultrasensitization was measured by plotting normalized peak response upon transient stimulation (i.e. the maximum of the time course) as a function of protein expression (see e.g. Fig. S2A). Additionally, the response coefficient of this peak response (similar to Eq. 12 in Protocol S1) was plotted as a function of an activation fraction (similar to Eq. 14 in Protocol S1) to allow direct comparison of ultrasensitization for different internalization kinetics (e.g. Fig. S2B; see also Legewie et al., 2005)

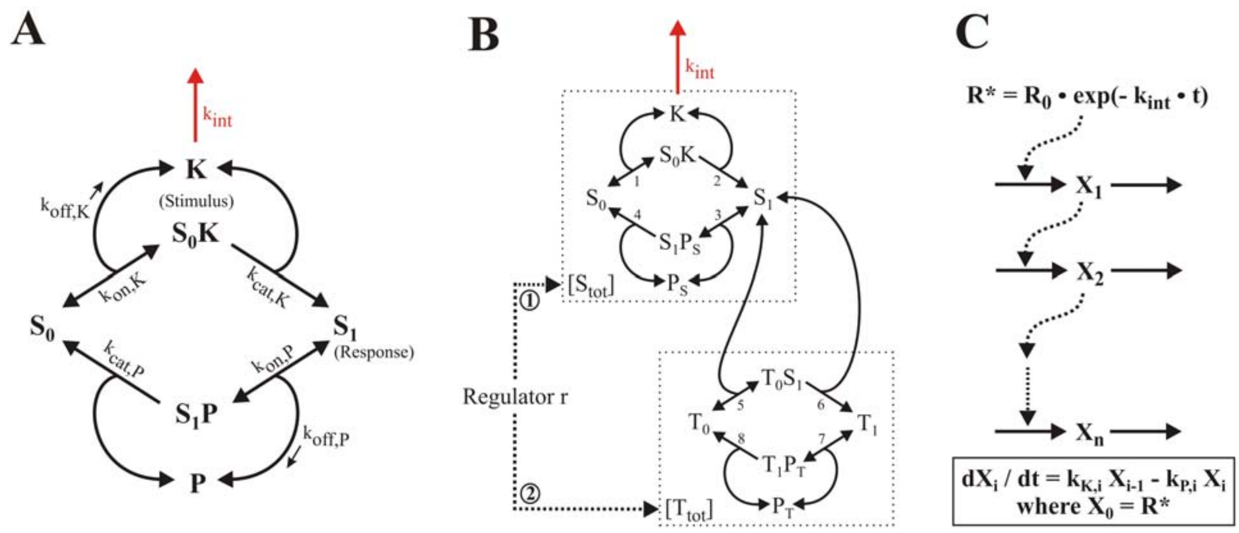

Fig. S1: Ultrasensitization upon transient stimulation

1) Ultrasensitization due to substrate sequestration:
Reanalysis of Fig. 3 in the paper upon transient stimulation reveals that the maximal normalized response, S_1 / S_{tot}, observed for strong substrate expression levels, S_{tot}, decreases with increasing internalization rates k_{int} (see Fig. S2A). This is due to the fact that under these conditions the stimulus duration is too short to elicit significant substrate phosphorylation. Nevertheless, ultrasensitization is preserved (albeit slightly weakened) even for such fast internalization rates as can be seen from Fig. S2B.

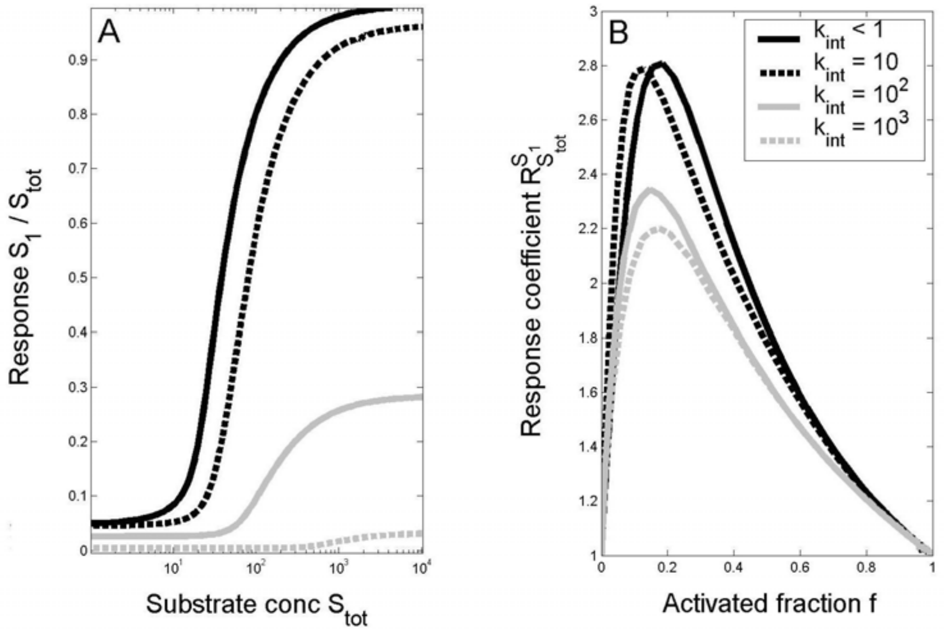

Fig. S2: Ultrasensitization due to substrate sequestration for varying internalization rates, k_{int} (Parameters chosen: $k_{on,k} = 1$; $k_{off,k} = k_{cat,k} = k_{off,p} = k_{cat,p} = 1$; $k_{on,P} = 2$; $K_{tot} = 100$; $P_{tot} = 10$)

Note: At a first glance it seems suprising that transient stimuli can elicit significant responses even for strong substrate expression levels, where the response time of a (de)phosphorylation cycle is very slow due to pronounced enzyme saturation (not shown). This is due to the fact that very high substrate expression levels, S_{tot}, result in sequestration of the kinase in the S_0K-complex and thereby delay internalization. Importantly, such delays are *not* significant in the range of ultrasensitization (not shown), i.e. the response coefficients plotted in S2B were obtained for transients with the half-life, $\tau \approx 1/k_{int}$. Similar conclusions also hold for Figs. S3 and S4.

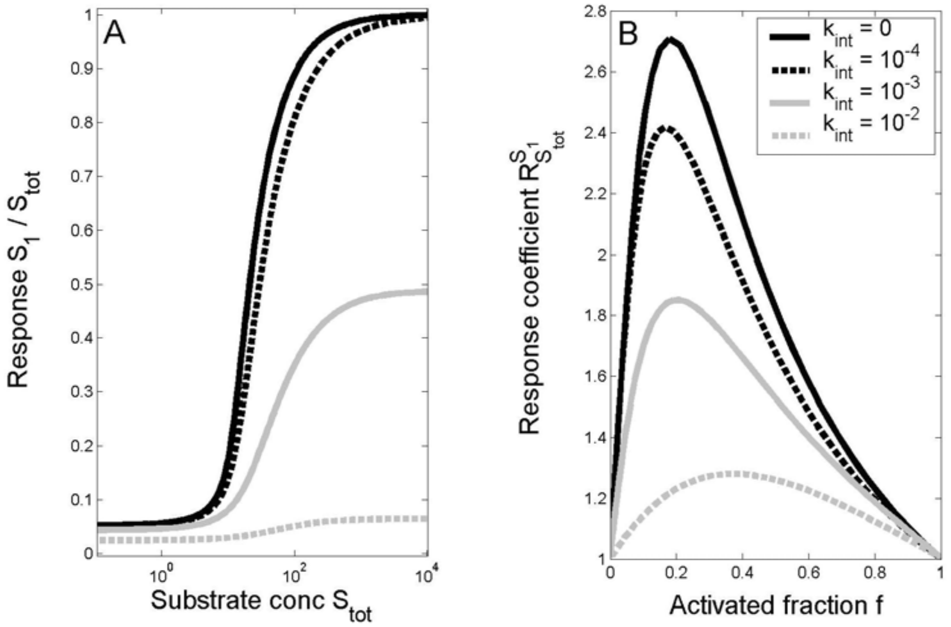

Fig. S3: Ultrasensitization due to activity switching for varying internalization rates, k_{int} (Parameters chosen: $k_{on,k} = 0.2$; $k_{off,k} = k_{cat,k} = k_{off,p} = 10$; $k_{cat,p} = 1$; $k_{on,P} = 20$; $K_{tot} = 1$; $P_{tot} = 1$)

2) Ultrasensitization due to activity switching:
Reanalysis of Fig. 4 in the manuscript upon transient stimulation (see Fig. S3) reveals that the maximal normalized response, S_1 / S_{tot}, again decreases for increasing internalization rates k_{int}., since stimulus duration is too short (see above). As shown in Fig. S3B, ultrasensitization due to activity switching is preserved (albeit weakened) as long as the stimulus duration is sufficiently long to elicit strong signal transmission.

3) Ultrasensitization due to synexpression within a kinase cascade:
Before analyzing ultrasensitization due to synexpression numerically, we shall discuss analytical results obtained by Heinrich et al. (2002) for a weakly kinase activated cascade upon transient stimulation. The corresponding model is shown in Fig. S1C, and the receptor (i.e. the input) time course was again modelled by a decaying exponential. For (an integral-based definition of) the transient amplitude, S_n, of a weakly activated cascade with n cascade stages Heinrich et al. (2002) derived:

$$S_n = \frac{R_0 \cdot \prod_{l=1}^{n} \frac{k_{K,l}}{k_{P,l}}}{\sqrt{1 + k_{int}{}^2 \cdot \sum_{j=1}^{n} \frac{1}{k_{P,j}{}^2}}} \qquad (1)$$

To analyze ultrasensitization due to synexpression in Eq. 1 we shall assume that an increase in the regulator, r (see Fig. 5 in the manuscript), leads to a proportional increase in all kinase (k_K) or all phosphatase (k_P) rate constants. According to the results obtained in Protocol S2, an m-fold change in the regulator, r, affects (non-normalized) steady state signal transmission upon weak stimulation in a cascade with n stages [m*n]-fold (see Eq. 10 in Protocol S2)

a) *The regulator, r, affects the expression of all kinases*: It can easily be seen from Eq. 1 that ultrasensitization due to synexpression of all kinases (where all $k_{K,i}$ are simultaneously altered by the regulator, r) is always preserved upon transient stimulation.

b) *The regulator, r, affects the expression of all phosphatases*: Ultrasensitization due to synexpression of all phosphatases (where all $k_{P,i}$ are simultaneously altered by the regulator, r) is also perfectly preserved as long as the condition $k_{P,i} >> k_{int}$ holds, since the Eq. 1 simplifies to:

$$S_n \approx R_0 \cdot \prod_{l=1}^{n} \frac{k_{K,l}}{k_{P,l}} \qquad (2)$$

As expected this result equals that observed at steady state for the constant input R = R_0, since the kinase cascade operates at steady state even for transient stimuli if the timescale of dephosphorylation is much faster than that of receptor internalization. By contrast, ultrasensitization due to synexpression of all phosphatases is weakened as soon as a single phosphatase operates on the same timescale as receptor internalization (i.e. as soon as a single $k_{P,i} \approx k_{int}$). In the extreme case, where all $k_{P,i} << k_{int}$, Eq. 1 reduces to:

$$S_n \approx \frac{R_0 \cdot \prod_{l=1}^{n} \frac{k_{K,l}}{k_{P,l}}}{\sqrt{\sum_{j=1}^{n} \frac{k_{int}^2}{k_{P,j}^2}}} = \frac{1}{r^{n-1}} \cdot \frac{R_0 \cdot \prod_{l=1}^{n} \frac{k_{K,l}}{K_{P,l}}}{\sqrt{\sum_{j=1}^{n} \frac{k_{int}^2}{K_{P,j}^2}}} \tag{3}$$

Here, we have used the relationship $k_{P,i} = r \cdot K_{P,i}$ to demonstrate that an m-fold change in the regulator, r, alters the transient amplitude, S, only $[(n-1) \cdot m]$-fold, so that ultrasensitization is slightly weakened when compared to the steady state result (see above).

The preceeding disccusion implies that ultradesensitization due to synexpression of all phosphatases is slightly weakened upon transient stimulation only for low levels of the regulator, r (i.e. low phosphatase expression), while ultrasensitization is perfectly preserved as the amount of regulator, r (i.e. phosphatase expression), is further increased.

Reanalysis of Fig. 5B in the paper upon transient stimulation (according to Fig. S1B) reveals that strong signal transmission in the cascade (i.e. $T_1 \approx T_{tot}$) occurs even for very short stimuli (i.e. large k_{int}), which is in contrast to the observations for a single phosphorylation-dephosphorylation cycle (see Figs. S1A, S2 and S3). This is due to the fact that even weak fractional activation levels of the upstream species, S (i.e. $S_1 << S_{tot}$), elicited by short transient inputs outweigh the phosphatase activity towards T for sufficiently large regulator levels, $r = S_{tot} = T_{tot}$, so that phosphorylation of T still occurs. Figure S4B confirms that ultrasensitization due to synexpression is preserved (albeit weakened) upon transient stimulation.

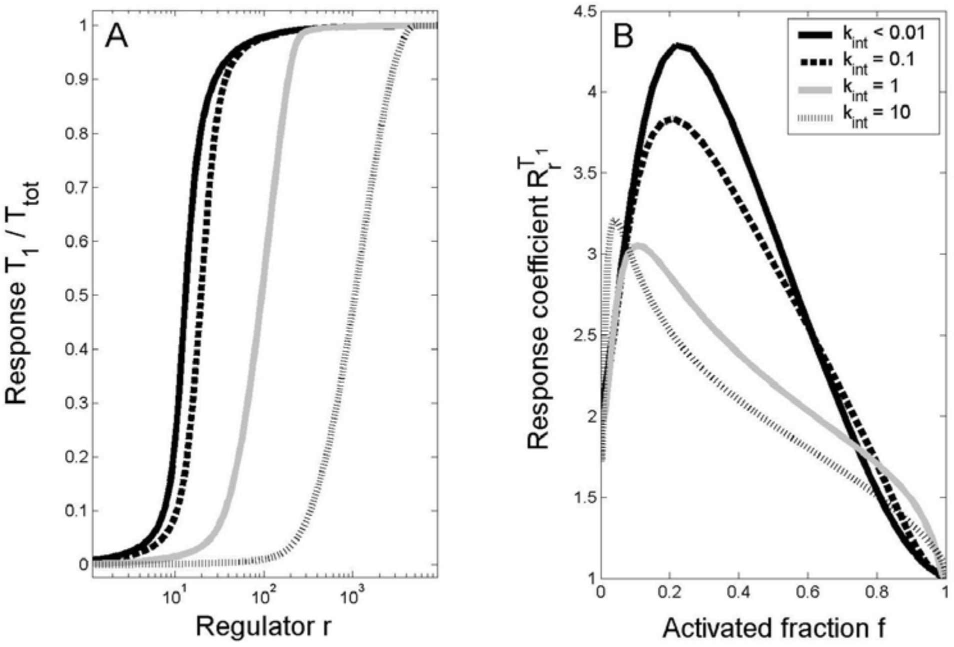

Fig. S4: Ultrasensitization due to activity switching for varying internalization rates, k_{int} (Parameters chosen: $k_{off,1} = k_{off,5} = k_{cat,2} = k_{cat,6} = k_{off,3} = k_{off,7} = k_{cat,8} = 1$; $k_{on,1} = 0.02$; $k_{on,5} = 0.2$; $k_{on,3} = 2.1$; $k_{on,7} = 2$; $k_{cat,4} = 1.1$; $K_{tot} = 10$; $P_{S,tot} = P_{T,tot} = 1$; $S_{tot} = T_{tot} = r$)

References

Heinrich R, Neel BG, Rapoport TA (2002) Mathematical models of protein kinase signal transduction. Mol Cell 9: 957-70.

Legewie S, Blüthgen N, Herzel H (2005) Quantitative analysis of ultrasensitive responses. FEBS J 272: 4071-4079.
